# Topological spatial representation in wild chacma baboons (*Papio ursinus*)

**DOI:** 10.1007/s10071-019-01253-6

**Published:** 2019-03-09

**Authors:** A. Louise de Raad, Russell A. Hill

**Affiliations:** 1 UHI Inverness College, University of the Highlands and Islands, 1 Inverness Campus, Inverness, IV2 5NA UK; 2 Department of Anthropology, Durham University, Durham, UK; 3 Primate and Predator Project, Lajuma Research Centre, Louis Trichardt, South Africa; 4 Department of Zoology, University of Venda, Thohoyandou, South Africa

**Keywords:** Spatial cognition, Primates, Animal movement, Change-point test, Route network, Navigation

## Abstract

Many species orient towards specific locations to reach important resources using different cognitive mechanisms. Some of these, such as path integration, are now well understood, but the cognitive orientation mechanisms that underlie movements in non-human primates remain the subject of debate. To investigate whether movements of chacma baboons are more consistent with Euclidean or topological spatial awareness, we investigated whether baboons made repeated use of the same network of pathways and tested three predictions resulting from the hypothesized use of Euclidean and topological spatial awareness. We recorded ranging behaviour of a group of baboons during 234 full days and 137 partial days in the Soutpansberg Mountains, South Africa. Results show that our baboons travelled through a dense network of repeated routes. In navigating this route network, the baboons did not approach travel goals from all directions, but instead approached them from a small number of the same directions, supporting topological spatial awareness. When leaving travel goals, baboons’ initial travel direction was significantly different from the direction to the next travel goal, again supporting topological spatial awareness. Although we found that our baboons travelled with similar linearity in the core area as in the periphery of their home range, this did not provide conclusive evidence for the existence of Euclidean spatial awareness, since the baboons could have accumulated a similar knowledge of the periphery as of the core area. Overall, our findings support the hypothesis that our baboons navigate using a topological map.

## Introduction

Despite diversity in morphology, ecology and behaviour, most animals need to orient and navigate towards specific places to reach important resources. Animals navigating through large-scale space face a complex environment in which they need to exploit resources. Remembering the locations of resources and travelling efficiently between them would thus be highly advantageous and directly affects animals’ survival and reproductive success (Normand and Boesch 2009). Studies often assume that efficient travel between goals results in straight-line travel and path linearity is frequently used to demonstrate the presence of efficient, goal-directed travel (Janson and Byrne 2007). For non-human primates, for example, routes between known resources often approximate straight lines (Cunningham and Janson 2007; Janson 1998; Normand et al. 2009; Valero and Byrne 2007). Yet, findings of goal-directed travel give little insight into the orientation mechanism that the animals may use during navigation.

Animals can navigate towards goals using different mechanisms. For example, path integration, where an animal continuously updates its position by integrating all its distances moved and changes of direction, has been reported in a wide variety of taxa (Trapanese et al. 2018). However, the mechanism is not precise and only enables animals to return to a certain start point after a single journey, and to rely on it solely results in navigational errors that become amplified further along the path the animal travels (Bennett 1996; Collet and Zeil 1998; Wehner 1992). Thus, while most animals use path integration in their movements (Etienne et al. 1998), they may also possess additional spatial cognitive abilities (Trapanese et al. 2018).

Path integration is often supplemented by route-based navigation that uses the topological relation between objects (Collet and Zeil 1998), a so-called topological map (Byrne 2000). Navigating the environment using a topological map envisions that an animal’s mental representation of locations and features in its environment consists of a set of interconnected, learned travel routes among sites (Di Fiore and Suarez 2007; Milton 2000; Poucet 1993). Topological maps thus represent the connectivity of the environments in a graph-structured network where intersections (also called vertices, nodes or junctions) represent well-defined locations (Scholkopf and Mallot 1995), such as food trees, at which animals make the decision about where to travel next (Di Fiore and Suarez 2007).

Alternatively, animals may possess highly detailed information about the spatial relationships among landmarks, which allows them to compute distance and direction from any one place to any other known place, based on a Euclidian representation of space (O’Keefe and Nadel 1978). True angles and distances between landmarks are represented within some kind of coordinate system, which allows an animal to compute routes between points that are out of view (Gallistel 1990). This quantitative representation of the environment is referred to as a Euclidean map or Euclidean spatial awareness (Bennett 1996) or coordinate-based map (Garber and Dolins 2014). Animals that possess Euclidean spatial awareness should travel by ‘computing’ a relatively straight or direct route to reach travel goals and have the ability to take novel routes and short cuts (Poucet 1993). In contrast, animals using a topological-based representation are expected to re-use the same set of tracks to reach travel goals that are located in the same part of their home range and to re-orient themselves at frequently used nodes or ‘decision-points’ (Suarez 2003). Topological maps have been argued to be an efficient system for storing environmental spatial information (Di Fiore and Suarez 2007; Poucet 1993) and less cognitively demanding than a Euclidean map because instead of remembering where resources are, animals have to associate the resources along familiar routes and memorise this association between landmarks and the nearby food sources (Bennett 1996; Garber 2000; Potì et al. 2005; Presotto and Izar 2010).

The distinctions between topological and Euclidean maps have allowed the precise cognitive mechanisms underpinning navigation to be tested in a number of species. Questions regarding spatial orientation and the mental representation of space have drawn particular attention in primate ecology (Boinski and Garber 2000; Garber and Dolins 2014; Janson 2016; Noser and Byrne 2014). Although numerous studies on spatial cognitive abilities in non-human primates have been conducted under controlled conditions in small-scale and simplified environments of captivity (Cramer and Gallistel 1997; Gibeault and MacDonald 2000; MacDonald and Agnes 1999; Menzel 1973; Potì 2000), the study of non-human primates’ navigational skills in their natural habitat has been relatively neglected (Janson and Byrne 2007); but see studies in Trapanese et al. (2018). In part this may be because crucial characteristics of a Euclidean map, such as the ability to take novel short cuts (Tolman 1948: but see; Noser and Byrne 2007a) and make detours and path innovations (Bennett 1996), are difficult to show in natural conditions where animals would rarely face a new environment (Janson 2000). Furthermore, a topological map with a very high number of landmarks is thought to be just as efficient as a Euclidean map (Byrne 2000; Janson and Byrne 2007). Discrimination between the different kinds of spatial representation becomes even more difficult where a given species could potentially use several mechanisms simultaneously (Lührs et al. 2009).

Despite these challenges, there is some evidence of Euclidean spatial awareness from primates in natural habitats (Gould 1986; Normand and Boesch 2009; Presotto and Izar 2010). Nevertheless, this has been questioned (Benhamou 1996; Bennett 1996; Byrne 2000; Janmaat et al. 2011; Poucet 1993) and there is now a growing body of evidence for topological spatial awareness in primates (Di Fiore and Suarez 2007; Erhart and Overdorff 2008; Milton 1980, 2000; Noser and Byrne 2007a, 2010; Presotto et al. 2018; Sigg and Stolba 1981). Use of a habitual route network has been reported for a number of primates (Boonratana 2000; Byrne 2000; Di Fiore and Suarez 2007; Erhart and Overdorff 2008; Hopkins 2011; Mackinnon 1974; Milton 2000; Noser and Byrne 2007a, 2010, 2014; Presotto and Izar 2010; Presotto et al. 2018; Schreier and Grove 2014; Sigg and Stolba 1981). Repeated use of particular tracks may be less linear than straight-line travel from one travel goal to the next, but can still have several advantages. For instance, repeated use of pathways could facilitate energy conservation by routing the animal according to particular landscape features (Di Fiore and Suarez 2007; Masello et al. 2017; Presotto and Izar 2010; Wilson et al. 2012). Such use of habitual routes would allow animals to forage efficiently by bringing them into contact with many potential feeding sources for monitoring or visitation (Di Fiore and Suarez 2007).

Although findings of repeatedly used travel routes are generally considered evidence that primates use a topological map, it is not necessarily evidence that they navigate (solely) using a topological map or lack a Euclidean spatial representation (Noser and Byrne 2007a; Presotto and Izar 2010). For instance, Presotto and Izar (2010) showed black capuchin monkeys (*Cebus nigritus*) did travel using habitual routes, but that they also travelled far from these habitual routes, and were thus not limited to a route-based network. Moreover, the monkeys could reach the same location from different starting points using different paths, even when they could not see a prominent landmark associated with that location and thus did not require continued sight of visible landmarks (Presotto and Izar 2010). Presotto and Izar (2010) concluded that the capuchins possessed topological spatial awareness but also some kind of Euclidean spatial awareness. Several studies have shown that baboons (*Papio* spp) use the shortest linear route to travel from one location to another and that they increase their travel speed as they approached out-of-sight water or food sources, indicating goal-directed and mental map processes (de Raad 2012; Noser 2004; Noser and Byrne 2007a, b, 2010; Pochron 2001, 2005; Sueur 2011). However, these findings do not allow discrimination between the types of orientation mechanism that baboons used during navigation.

In this paper, we investigate whether movements of chacma baboons (*Papio ursinus*) are more consistent with topological or Euclidean spatial awareness. First, baboon travel routes were investigated to determine whether baboons use a route network to navigate through the landscape. However, in light of arguments that the use of habitual routes does not necessarily exclude a Euclidean map-like awareness (Presotto and Izar 2010), three predictions resulting from the hypothesized use of Euclidean maps and topological maps were tested to discriminate between these navigation mechanisms (Table 1). Although these predictions are unable to confirm the existence of a Euclidean spatial awareness, each prediction is able to provide strong support for the existence of topological spatial awareness. Furthermore, although each prediction by itself might not conclusively discriminate between the two different kinds of spatial representation, the three predictions combined may provide a clear support for one or the other alternative.

**Table 1:** Three predictions resulting from the hypothesized use of Euclidean and topological spatial awareness along with the support provided by this study

	Euclidean spatial awareness	Topological spatial awareness
Prediction 1: Travel route linearity	There will be no significant difference in travel route linearity between the core area and peripheral area of baboons’ home range (both being highly linear)	Travel route linearity will be higher in the core area than in the peripheral area of baboons’ home range
	Partially supported	
Prediction 2: Approaching travel goals	Baboons will arrive at travel goals from all possible directions	Baboons will approach each travel goal from the same or a small number of direction(s)
		Supported
Prediction 3: Leaving travel goals	There will be no significant difference between the “initial leaving direction” when leaving a travel goal and the “general leaving direction” to the next travel goal	There will be a significant difference between the “initial leaving direction” when leaving a travel goal and the “general leaving direction” to the next travel goal
		Supported

Prediction 1: If baboons navigate using Euclidean spatial awareness, their navigation should remain efficient even in lesser-known, peripheral areas of the home range (following Gallistel and Cramer 1996; Normand and Boesch 2009). In contrast, differences in movement patterns between the baboons’ core area and peripheral areas are expected when navigating using a topological map, as the further they move from the core area the fewer available familiar landmarks (particularly topographical features) they have to guide their movement (Normand and Boesch 2009). Thus, if navigation is significantly less linear in the periphery than in the well-known core area, this would provide evidence for the use of a topological map.

Prediction 2: If baboons navigate by a Euclidean spatial awareness this allows them to arrive at known goals from multiple directions approximating a random distribution of approach angles, whereas if navigating using a topological map, they would be more likely to approach a travel goal from a small number of the same direction(s), using the same landmarks or routes every time.

Prediction 3: If baboons are using a Euclidean spatial awareness to navigate, the initial direction adopted when leaving a travel goal should not significantly differ from the general direction to the next goal, since animals would know the exact direction of the next goal and travel in a goal-directed manner (Normand and Boesch 2009). In contrast, if animals travel using landmarks, the difference between these two directions is expected to be higher because animals would have to reorient along the way when encountering landmarks or nodes (Di Fiore and Suarez 2007).

It has been suggested that animals may plan further ahead when foods are limited and as such that cognitive mechanisms may become more evident during the dry winter season (Valero and Byrne 2007). For this reason, we also analysed winter and summer data separately.

## Methodology

### Study species and site

Chacma baboons (*Papio ursinus*) are large, terrestrial primates (adult males, 17–30 kg; females, 10–15 kg); Barrett and Henzi (1997) that live in large, multi-male, multi-female groups, ranging between 4 and 128 individuals (Bettridge et al. 2010). Baboons have large home ranges and day ranges compared to other primate species making them an ideal subject to study movement and spatial awareness at a large-scale space.

We conducted fieldwork at Lajuma Research Centre in the Soutpansberg Mountains, Limpopo Province, South Africa (23°02′23″S, 29°26′05″E) between April 2007 and December 2008. The study site ranged in elevation from 1150 to 1750 m (Willems and Hill 2009). The mountains have many topological features, such as cliffs and mountain tops that may have aided baboons in navigation. In particular, the highest peak of the mountain range (Letjume) was visible from many locations within the baboons’ home range and was likely to serve as a prominent landmark. Furthermore, many man-made tracks and natural game trails were present throughout the study area and junctions in such tracks could also serve as navigational landmarks.

Significant local variation in abiotic factors such as water availability and elevation results in a variety of microclimates that support a substantial diversity of both flora and fauna (Berger et al. 2003). Vegetation is categorised as a complex mosaic of habitat types classified under the Soutpansberg mistbelt forest group (von Maltitz 2003) and includes small, fragmented patches of tall mistbelt forest and riparian forest separated by woodlands, thickets, grasslands and peatlands (Mucina and Rutherford 2006). Clear habitat boundaries were difficult to define, however, and the baboons predominantly foraged along a woodland-thicket-grassland gradient. Leopards (*Panthera pardus*) were a significant predator on site (Chase Grey et al. 2017) with large raptors (African crowned eagle, *Stephanoaetus coronatus*; African black eagle, *Aquila verreauxii*) and African rock pythons (*Python sebae*) also present (Coleman and Hill 2014; Willems and Hill 2009). Local climate is classified as temperate/mesothermal, with cool dry winters from April to September and warm to hot wet summers from October to March; mean annual temperature averages 17.1 °C, with a mean annual rainfall of 724 mm (Willems et al. 2009).

### Behavioural observations

House group contained approximately 60 individuals and was one of the largest of at least five baboon groups ranging across the study area. The group was habituated to human researchers with individual baboons observed at distances of approximately 10 m from within the group. We collected data for 234 full follow days (from their morning sleeping site to their evening sleeping site) and 137 partial days. During follows, track points (*N* = 462,556) were automatically recorded on a Garmin GPSMAP60CSx, resulting in a track point on average every 5.35 m (± 4.87 m) and an average time lapse between consecutive track points of 23 s (± 44 s). Track points thus reflected the group’s movement rather than individual trajectories within the group. We identified 12 different sleeping sites within the baboons’ home range during our full follow days (de Raad 2012).

Data were processed using Hawth’s Analysis Tool 3.26 (Beyer 2004) in ArcMap 9.3 (ESRI 2010) so that consecutive track points were exactly 20.0 m apart (de Raad 2012) to (1) reduce the errors in the representation of baboon movements caused by observer movement within the troop while recording other behavioural data, (2) remove standstill GPS accuracy errors and (3) remove large clumps of track points at locations where the troop was more or less stationary for long periods of time, such as near sleeping sites.

### Route network

To determine whether baboons travelled through a network of habitual routes within their home range we applied a method devised by Di Fiore and Suarez (2007). This entailed overlaying all recorded tracks (*N* = 317) in ArcMap 9.3 (ESRI 2010) and then identifying, by eye, all tracks that appeared to have been used more than once. These ‘initial routes’ were sketched and digitized using the ‘Editor Tool’ and were then confirmed by superimposing, one at a time, the individual tracks. We included an initial route into the habitual route network when baboons followed the same trajectory, defined as within 25 m, for at least 100 m on at least four different days (Di Fiore and Suarez 2007; Presotto and Izar 2010). In subsequent analyses, we applied a more stringent criterion where an initial route would only be included into the habitual route network if the trajectory was traversed on at least ten different days. To determine whether baboons ranged more often in proximity to the route network compared to other areas we then overlaid the recorded track points (after application of the 20 m filter) with the resulting habitual route networks and using a ‘Spatial Join’ in ArcMap 9.3 estimated the proportion of track points that fell within 5 m, 10 m, 15 m, and 25 m buffers around the route network (defined using the ‘Multiple Ring Buffer’ tool in ArcMap 9.3).

Where two or more routes within the habitual route network crossed (or joined), the location was defined as an intersection. Intersections may simply be an arbitrary junction of routes or could represent potential decision points where baboons decide where to travel next, being free to choose to turn down any of the intersecting routes (Di Fiore and Suarez 2007). To test whether route intersections were indeed decision points, two approaches were used.

First, following Di Fiore and Suarez (2007), each track was overlaid on the habitual route network map one at a time and the approach and leaving directions at each intersection were tallied. When at least two alternate tracks were selected for a particular intersection from a single approach direction, or when the same track was taken at an intersection following approaches from different directions, the intersection was scored as a decision point (see Di Fiore and Suarez 2007). The second approach examined the spatial proximity between route intersections and ‘change-points’ (see below). Analyses were carried out in ArcMap version 9.3 (ESRI 2010).

### Travel goals

Locations where baboons significantly changed their direction of travel were considered travel goals. To identify travel goals, we used the change-point test (CPT) (Byrne et al. 2009) on the 234 full follow days in R software (R Core Team 2016). The CPT is based on the statistical characteristics of a subject’s daily track, to circumvent the problem that researchers cannot know in advance the goal of the subject whose ranging behaviour is being recorded. In summary, the CPT compares whether a set of vectors before a waypoint (the potential “change-point”) in an animal travel route (*R*_k_) is collinear with a set of vectors after that waypoint (*R*_q_) (after and before are from the “travel direction point of view”) whereby the lengths of distance vectors *R*_q_, *R*_k_, and the length of the resultant vector *R*_(q+k)_ are used as indicators for collinearity (see Byrne et al. 2009 for details). If *R*_q_ and *R*_k_ are collinear, then the test is re-applied at the next waypoint, but when *R*_q_ and *R*_k_ depart from collinearity at the pre-set level of significance (α), a change-point is identified (Noser and Byrne 2014). The value of ‘*q*’ (the number of vectors before the potential change-point) has to be determined by the user in advance and remains the same throughout the use of the CPT. Two distinct features of the CPT are (1) it is sequentially applied to segments of travel “backwards in time” and so the test starts at the end of the track, and (2) that once a waypoint is identified as a change-point, this location then becomes the starting point for the second iteration of the CPT and so on (i.e. the CPT must be re-applied each time after a change-point is identified) (Byrne et al. 2009). To effectively run the CPT on our large dataset, we have updated the original R script provided by Byrne et al. (2009) to include both a ‘day loop’ and a ‘*q*-value loop’ (de Raad, Stephens, Tomlin, Barton and Hill, in preparation), removing the need to re-run the test every time a change-point has been identified.

**Fig. 1:**
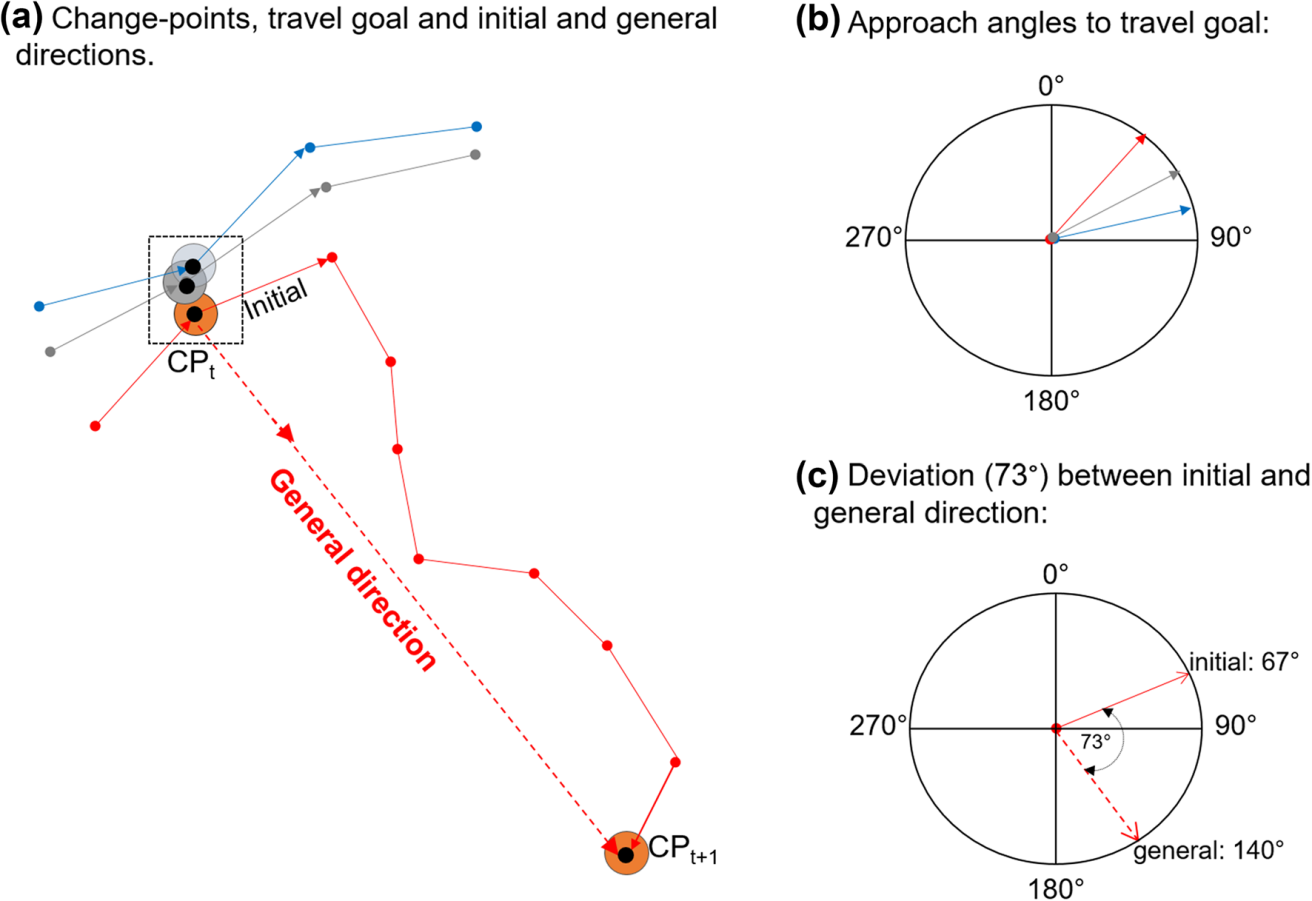
**a** Change-points (black points) identified by the CPT were buffered by a 10 m buffer (blue, grey and red circles) and when these buffers overlapped (e.g. the 3 CP within the black dashed box), they were considered to be one travel goal. If change points did not overlap each was considered a separate travel goal (e.g. CPt + 1). The initial travel direction was the direction of the first step after leaving a change-point (from a track-point identified as a change-point to the next track point) and the general direction is that of one change-point (CPt) to the next (CPt + 1) (here shown for the red travel route only). **b** Approach angles were identified for each travel goal separately (here *N* = 3). **c** Deviation (73°) was analysed as the difference between the initial leaving direction (67°) and the general direction (140°)

The CPT thus identifies change-points, locations at which animals start orienting towards the next travel goal, which in the majority of cases can be readily interpreted in biological terms (Asensio et al. 2011; Ban et al. 2016; Byrne et al. 2009; Cunningham and Janson 2013; Howard et al. 2015; Janmaat et al. 2011; Joly and Zimmermann 2011; Noser and Byrne 2007a, 2014; Presotto et al. 2018). We applied a 10 m buffer to each change-point and highly clumped change-points with overlapping buffer areas were treated as the same travel goal (Fig. 1a) (e.g. a fruiting tree can be an important resource for an extended period at which location baboons would change their travel direction; a slightly different change-point would be identified in close proximity to the tree on multiple days, each reflecting the same location). Travel goals are, therefore, either individual change-points or highly clumped change-points representing the same travel goal, and analyses were carried out for each unique travel goal.

### Prediction 1: Ranging and path linearity

To investigate differences in movement patterns between an animal’s core area and peripheral areas, we estimated the baboons’ home range from track points using the adaptive Local Convex Hull (LoCoH) method (Getz et al. 2007) at the 99% isopleth level with a value of *a* = 3000 (de Raad 2012) in R software (R Development Team 2016). The core area was subsequently defined at the 75% isopleth level (following Normand and Boesch 2009) and peripheral areas were defined as the difference between the home range and the core area (i.e. the difference between the 99% and 75% isopleth levels).

Path segments were defined as ‘travel between consecutive travel goals’ and only path segments with a minimum of six track points (i.e. 5 steps) and path segments that fell entirely within the core area (*N* = 200) or entirely with the periphery (*N* = 301) were included in this analysis. Path segments had an average beeline distance (i.e. beeline distance between two consecutive change-points) of 162.0 m and an actual distance travelled (the sum of individual steps) of 246.7 m, which was considered to be large enough to avoid the bias of short path segments which could, in the case of a topological map, be linked to too few landmarks (Normand and Boesch 2009).

Linearity was calculated for each path segment using a linearity index computed as the ratio between the beeline distance (*D*) of the path segment and the actual track length travelled (the sum of individual step lengths) [the *R* value: Batschelet (1981)]. Linearity indices ranged between 0 and 1 and the closer the value approaches 1, the smaller the angular deviation of the vectors and thus the more linear the corresponding path segment (Batschelet 1981).

### Prediction 2: Approach directions

To investigate whether baboons arrived at travel goals from the same direction or from all possible directions we first identified those tracks that intersected with a travel goal using ‘Spatial Joins’ in ArcGIS 9.3 (ESRI 2010) between tracks and travel goals. Travel goals identified for summer were joined only to summer tracks and travel goals identified for winter only to winter tracks.

For each travel goal, we determined the direction of the final step approaching the travel goal as a compass direction (deviation from the True North) between 0° and 360° (Di Fiore and Suarez 2007; Normand and Boesch 2009). This was done based on the coordinates of the track point at the start of the final step and the coordinates of track point at the end of that step (i.e. the coordinates of the identified change-point) using the circular statistics software program Oriana (Kovach Computing Services 2009) (Fig. 1b). Due to the potential impact of small sample sizes on the results, only travel goals with a minimum of 15 approaches (*N* ≥ 15) were included in this analysis (de Raad 2012). To examine the distribution of final approach directions around the circle, we performed a parametric Rao’s spacing test on each travel goal in Oriana. Rao’s spacing test takes as its null hypothesis that the data are uniformly distributed. For a uniform distribution, the spacing between points should be roughly 360°/n. If the actual spacing deviates too much from this value then the likelihood that the data are uniformly distributed is reduced. Rao’s spacing test can be more powerful than the commonly used Kuiper’s *V* test or Rayleigh test (by e.g. Valero and Byrne 2007), especially when the data are bimodal (Kovach Computing Services 2009). Non-parametric Watson’s U2 test and Kuiper’s test produced similar results confirming the robustness of the analyses (de Raad 2012).

### Prediction 3: Leaving directions

The ‘initial leaving step’ was identified as travel between a travel goal and the first track point after that travel goal whereby the ‘initial leaving direction’ was calculated based on the coordinates of the travel goal and the first track point after that travel goal using the circular statistics software program Oriana (Kovach Computing Services 2009) (Fig. 1a). ‘General leaving direction’ was identified as the direction from one travel goal to the next (Fig. 1a). Both initial and general leaving directions were calculated as a compass direction (deviation from the True North) between 0° and 360° (Di Fiore and Suarez 2007; Normand and Boesch 2009) (Fig. 1c).

We investigated whether initial leaving directions significantly differed from the general leaving directions by examining the deviation between the two (Fig. 1c) (Janson 1998). If baboons would know the precise direction towards the next goal, their travel route is expected to resemble straight-line travel. Under the Euclidean map hypothesis, the deviation between the initial and general leaving directions was thus expected to approach zero. GPS accuracy had to be taken into account since this may influence the expected deviation from a straight line. Although GPS error was small and usually less than 8 m (personal observation) it was not formally determined in this study. We, therefore, accounted for GPS accuracy using the more conservative GPS error value of 14.2 m determined by Normand and Boesch (2009) who used the same model of handheld GPS, to accommodate the potential impact of dense canopy and cliff faces/topography on GPS accuracy in certain locations. For a distance travelled of 50 m, for example, the consequences of the inaccuracy of the GPS (14.2 m) to measure the correct angle would be 15.82° and the linearity index would become 0.9619 instead of 1 theoretically for a straight line (Normand and Boesch 2009). We standardised deviation values to between 0° and 180° resulting in a linear variable that could be analysed using linear statistics, as done by (Normand and Boesch 2009). To ensure the data approximated to a normal distribution, a third-root transformation ([deviation]^1/3^) was applied (Zar 1999). Therefore, deviation was considered significantly different from a straight line if it was larger than 15.82°. For an average step length of 50.2 m, this could lead to a maximum of 15.82° error in the angle estimation. Subsequently, one-sample paired *T* tests were performed in PASW Statistics release version 17.0.0 (SPSS Inc. 2008), with an expected value of 2.51 ([15.82]^1/3^).

## Results

### Route network

**Fig. 2:**
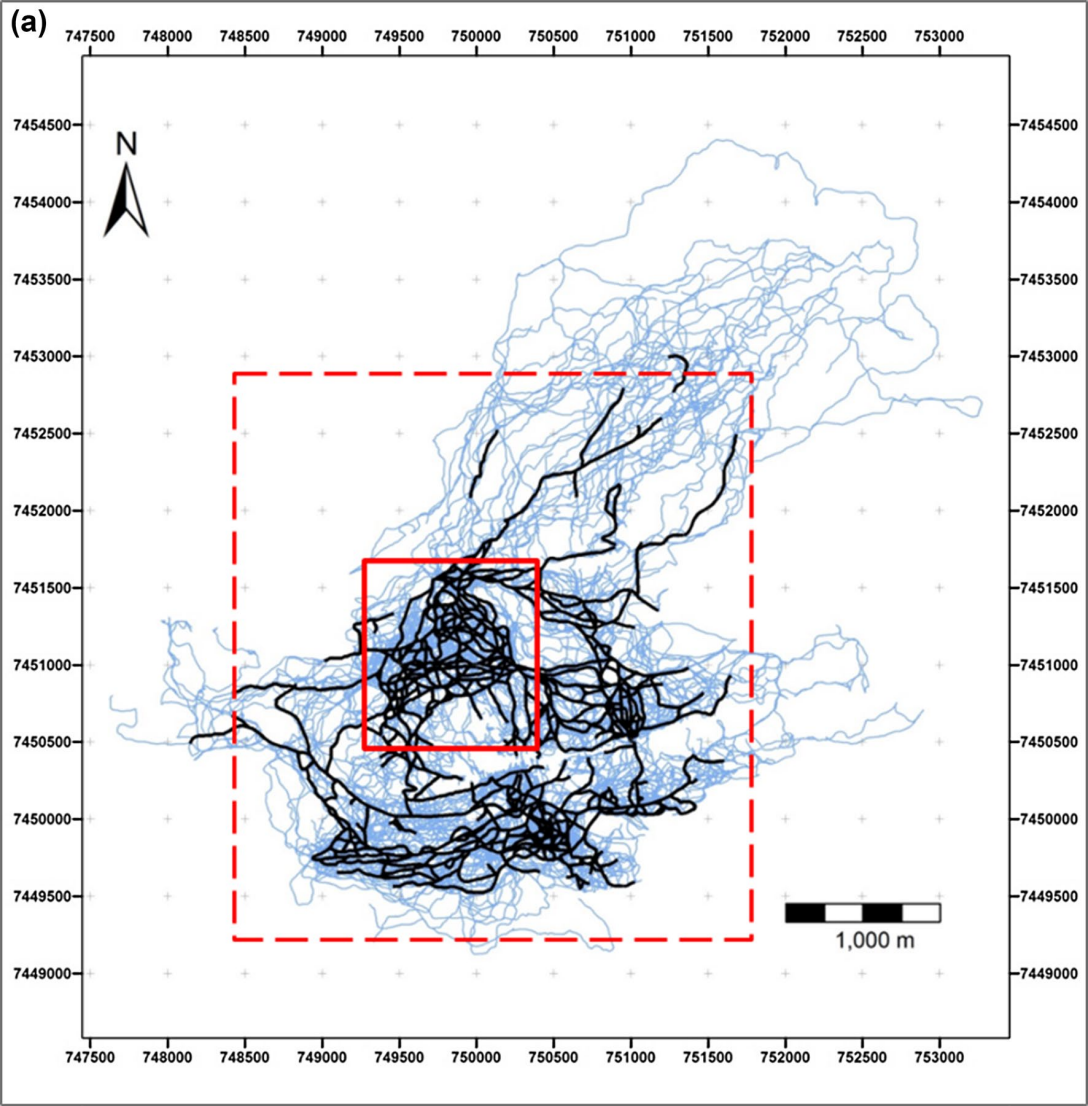

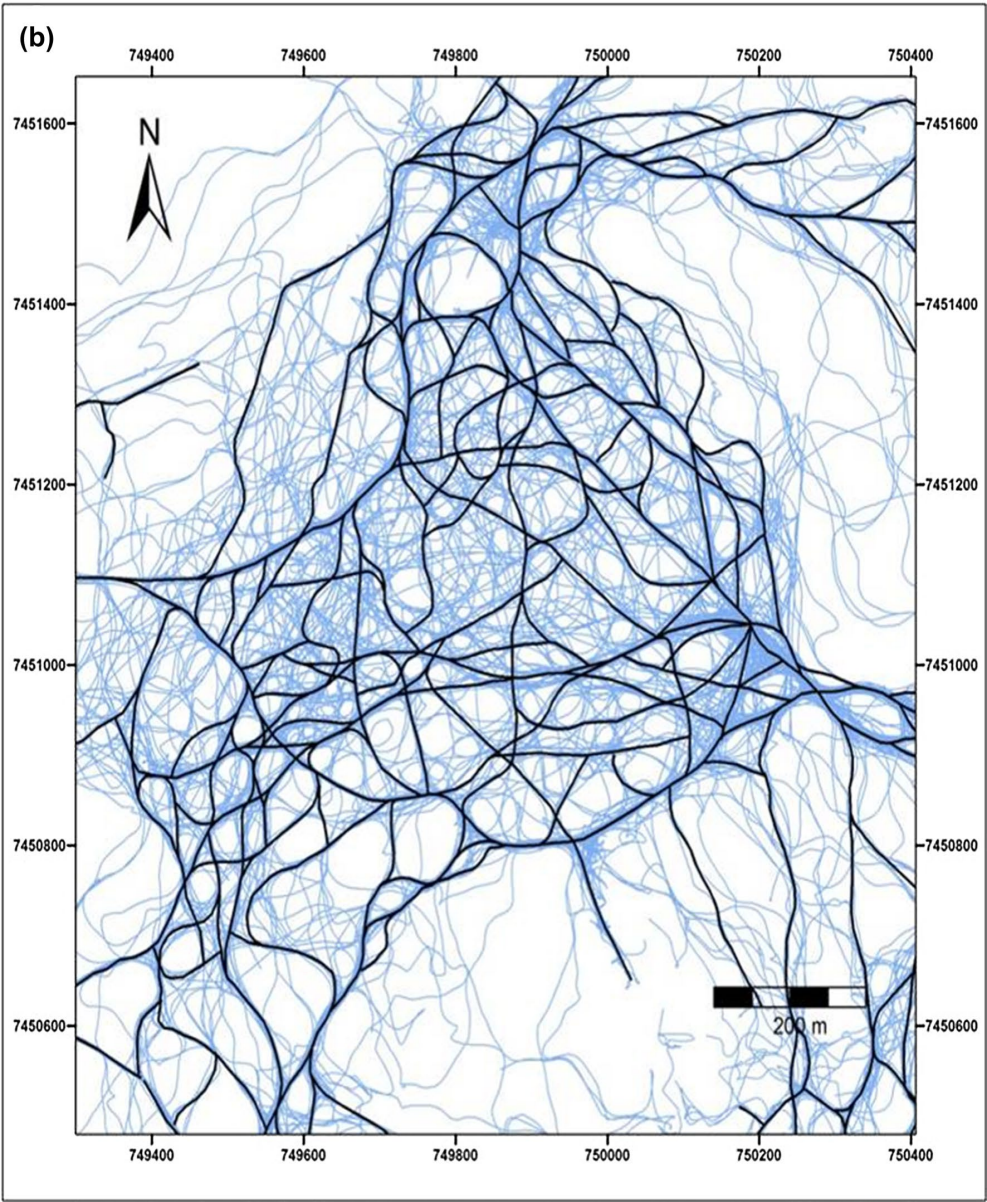

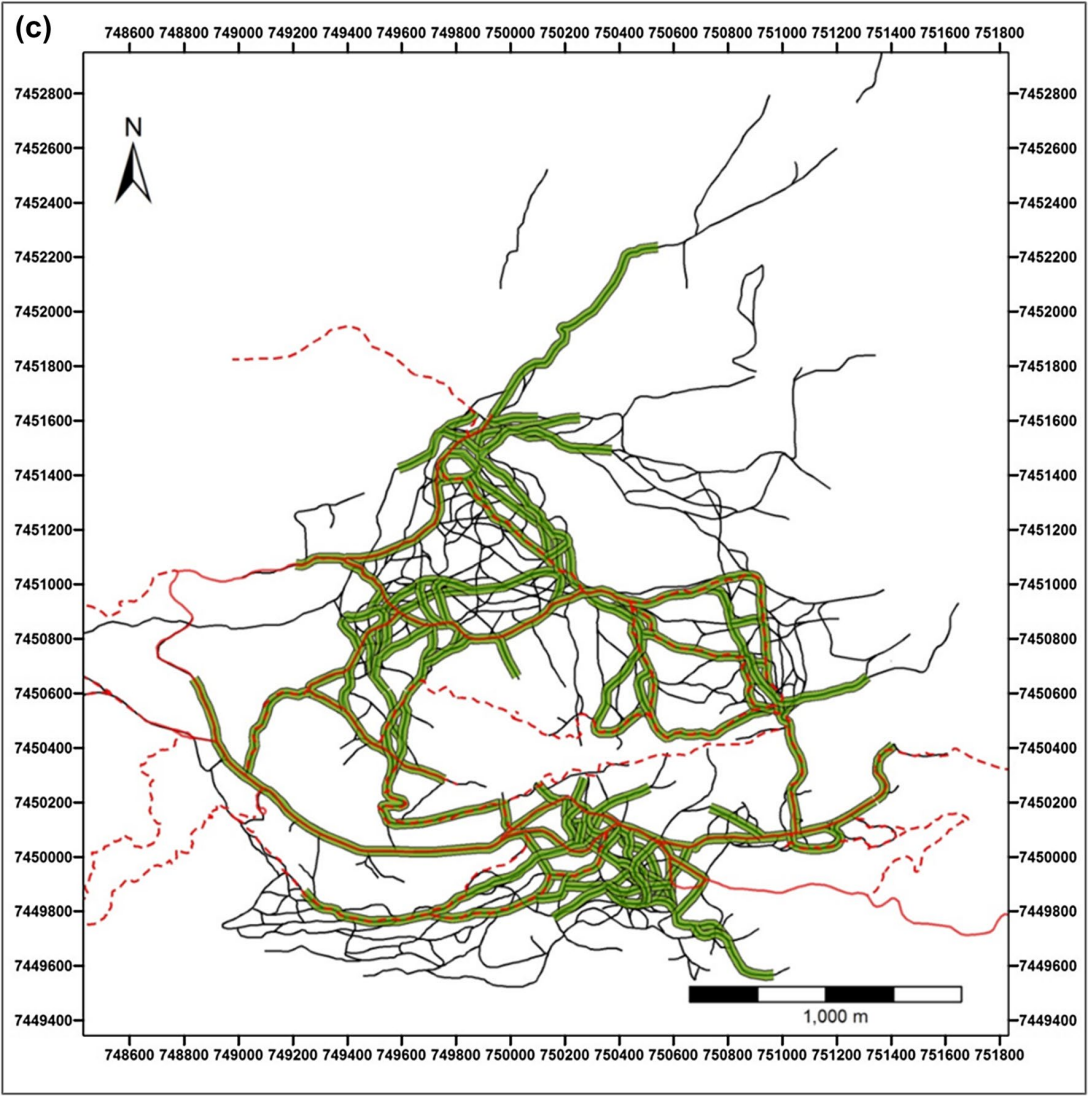
**a** Tracks (*N* = 371) (fine blue lines) overlaid with the network of habitual routes (black lines; based on the ‘4 repetition criteria’). Boxed areas represent the extent of **b** (solid red line) and **c** (dashed red line). **b** Tracks (fine blue lines) in part of the baboons’ home range overlaid with the habitual route network (black lines; based on the ‘4 repetition criteria’). **c** Baboons’ habitual route network based on the 4-day criteria (black lines) and the 10-day criteria (thick green lines). Many elements of the ‘highway’ network created with the 10-day criteria correspond to man-made tracks (red lines) or game trails (red dashed lines)

Tracing of habitual paths revealed a dense network of repeated routes (on at least 4 days) spread throughout the group’s home range (Fig. 2a, b). Track points (*N* = 462,556) did not fall equally within the 5 m buffers around the habitual network (total track points: *df* = 4, *χ*^2^ = 113593.6, *p* < 0.001) (Table 2). With 79.5% of track points within 25 m of the route network and more than 50% of all location records within 5 m, the baboons ranged significantly more in close proximity to the route network. We identified 657 intersections in the route network. Consistent with the idea of a topological map, 86% (*N* = 565) of intersections were also confirmed as decision points. Moreover, 42% (*N* = 268) of all intersections were found at the same location as a change-point and 92% (*N* = 589) of intersections were located within 50 m of a change-point.

**Table 2:** Percentage of all track points (*N* = 462,556) that fell within the different bands around the habitual route networks using the 4 days criteria

Buffer	4-day criteria network (%)
0–5 m	53.6
5–10 m	13.3
10–15 m	5.9
15–20 m	3.9
20–25 m	2.7
Total (0–25 m)	79.5

Parts of the habitual route network were used on more than 10 days and some on more than 50 days. When we applied a more restrictive criterion of 10 or more repetitions to identify those parts of the habitual route network used more intensively, this revealed a network of ‘highways’ (Fig. 2c) that were often associated with man-made tracks or game trails. Under this more stringent criterion, 56% (*N* = 259,031) of all location records still fell within 25 m of the highway network.

### Travel goals

The CPT identified 1058 change-points throughout the baboons’ home range for the 234 full follow days. For summer (*N* = 441 change-points), 79 change-points were grouped into 45 travel goals resulting in 407 summer travel goals. For winter (*N* = 617 change-points), 172 change-points were grouped into 71 travel goals resulting in 516 winter travel goals.

### Prediction 1: Travel route linearity between core area and periphery

**Fig. 3:**
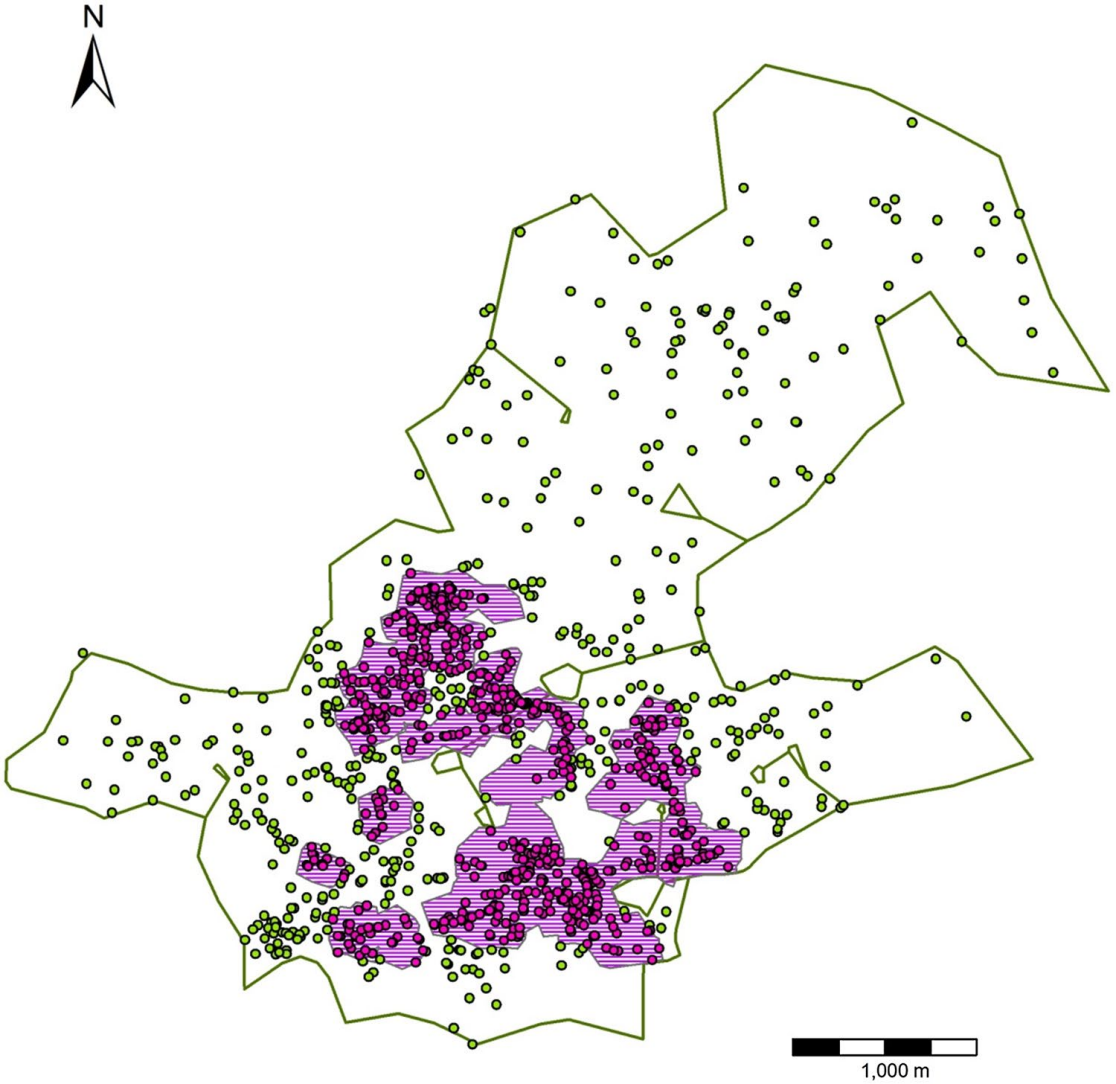
Home range boundary (green line) and core area (purple striped area) delineated by 99% and 75% isopleths respectively, estimated using the adaptive Local Convex Hull (a-LoCoH) method (Getz et al. 2007) with *a* = 3000. Change-points in the core area (purple dots) and in the periphery (green dots) are shown

Home range size of our study group was 12.4 km^2^ with a core area of 2.0 km^2^ (Fig. 3). The linearity of path segments in the core area (*N* = 200, median LI = 0.815) was not significantly different from the linearity of path segments found in peripheral areas (*N* = 301, median LI = 0.808) (Mann–Whitney *U* test: *U* = 29352.0, *Z* = − 0.471, *p* = 0.637). Furthermore, we found no significant difference in actual distance travelled between subsequent travel goals between the core area and the periphery (*U* = 29051.0, *Z* = − 0.661, *p* = 0.509) or straight-line distance between subsequent travel goals between the core area and the periphery (*U* = 28888.0, *Z* = − 0.764, *p* = 0.455). Although this does not support the sole use of a topological map, it also does not provide conclusive evidence for the existence of Euclidean spatial awareness, since the baboons could have accumulated a similar knowledge of the periphery as of the core area.

Across the home range, baboons showed significantly more direct travel in winter (*N* = 467, LI = 0.820) than in summer (*N* = 362, LI = 0.8000) (Mann–Whitney *U* test: *U* = 77274.0, *Z* = − 2.121, *p* = 0.038). Nevertheless, there was no significant difference in the linearity of path segments between core area (*N* = 92, median LI = 0.803) and peripheral areas in winter (*N* = 133, median LI = 0.7742) (Mann–Whitney *U* test: *U* = 5618.0, *Z* = − 1.042, *p* = 0.298).

### Prediction 2: Approach directions

**Fig. 4:**
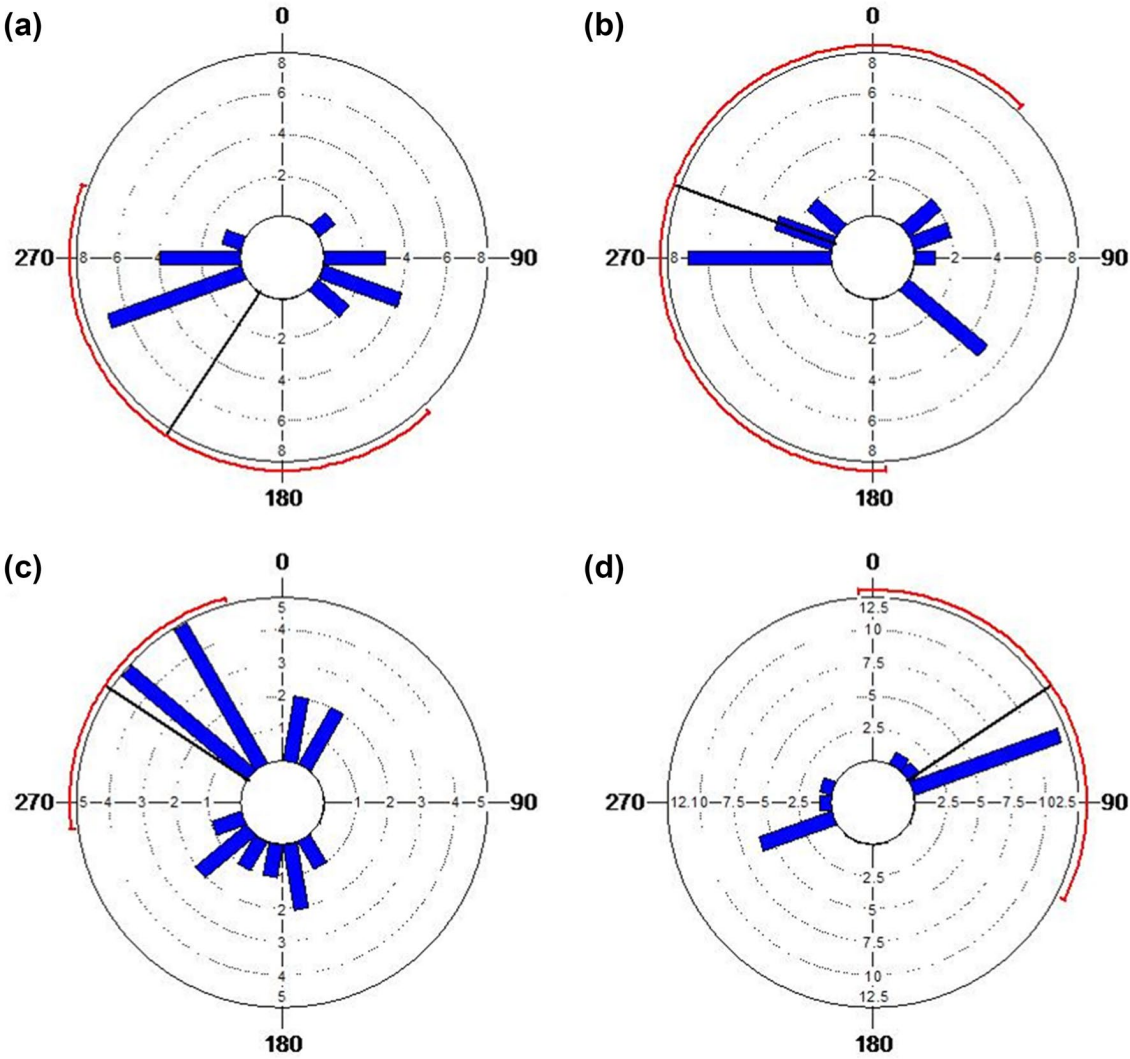
Distribution of approach angles for two selected travel goals for summer: **a** SUM-783 and **b** SUM-877, and two selected goals for winter **c** WIN-11 and **d** WIN-235. Note that the parallel side bars show the number of observations within each class range (width of class range is 10°), but that the linear scale of the axis varies between resources (for **a, b** each dotted circle represents 2 observations, for **c** each dotted circle represents 1 observation and for **d** each dotted circle represents 2.5 observations)

We analysed the approach directions for 17 summer travel goals and 17 winter travel goals which had at least 15 approaches (Table 3). For the great majority of these travel goals (*N* = 29, 85.3%), the approach angle distributions were significantly clumped (Rao’s Spacing test, *P* < 0.01) (Fig. 4) and only two of the winter travel goals had approach angles that were randomly distributed around the circle. Overall, this result suggests that baboons do not approach the travel goals from all directions and instead approach them from consistent direction(s), providing strong support for the topological map hypothesis.

**Table 3:** Analysis of the distributions of approach angles for 17 summer and 17 winter travel goals using Rao’s Spacing test (with *U* and *p* values shown)

	*N*	*µ*	*r*	*U* value	*p* value
SUM-526	19	129.4	0.10	274.3	< 0.01
SUM-532	15	243.3	0.47	196.4	< 0.01
SUM-556	15	224.9	0.20	193.2	< 0.01
SUM-566	18	315.8	0.37	299.1	< 0.01
SUM-611	15	51.8	0.25	196.2	< 0.01
SUM-647	16	253.1	0.09	195.7	< 0.01
SUM-654	27	225.3	0.12	201.1	< 0.01
SUM-656	15	267.4	0.22	250.2	< 0.01
SUM-672	16	280.3	0.62	288.8	< 0.01
SUM-676	24	300.7	0.47	176.7	< 0.01
SUM-717	20	287.3	0.63	216.5	< 0.01
SUM-741	20	104.1	0.21	242.0	< 0.01
SUM-754	20	329.9	0.51	229.5	< 0.01
SUM-783	22	213.3	0.22	227.5	< 0.01
SUM-804	24	356.2	0.36	228.5	< 0.01
SUM-805	16	239.5	0.70	220.2	< 0.01
SUM-877	22	290.4	0.15	225.7	< 0.01
WIN-11	22	303.7	0.40	171.0	< 0.05
WIN-34	39	185.8	0.15	220.5	< 0.01
WIN-75	15	84.3	0.33	266.1	< 0.01
WIN-76	25	348.5	0.30	245.4	< 0.01
WIN-94	23	301.7	0.12	273.6	< 0.01
WIN-157	15	315.9	0.31	224.7	< 0.01
WIN-206	15	65.7	0.53	239.6	< 0.01
WIN-208	41	178.2	0.52	182.4	< 0.01
WIN-222	15	256.0	0.45	202.9	< 0.01
WIN-235	22	56.3	0.28	239.0	< 0.01
WIN-252	16	37.9	0.11	250.1	< 0.01
WIN-295	18	316.2	0.22	240.6	< 0.01
WIN-306	15	6.0	0.10	259.5	< 0.01
WIN-424	15	289.3	0.31	284.2	< 0.01
WIN-426	17	241.2	0.43	219.1	< 0.01
WIN-440	16	269.6	0.31	138.3	ns
WIN-454	15	262.0	0.35	164.5	ns

For each resource, sample size (*N*), mean approach angle (*µ*) and length of the mean vector (*r*) are shown

### Prediction 3: Leaving directions

The deviation between the initial leaving direction and general direction was significantly different from expected straight-line travel when taking GPS error into account, both for the year as a whole (one-sample *t* test: *t* = 8.666, *df* = 827, *p* < 0.001) and for summer (*t* = 5.491, *df* = 360, *p* < 0.001) and winter (*t* = 6.703, *df* = 466, *p* < 0.001) separately. There was no significant difference in mean deviation between initial and general direction between summer and winter (*t* test: *t* = 0.369, *df* = 780, *p* = 0.712). These results suggest that baboons lack Euclidean spatial awareness.

## Discussion

Chacma baboons within the Soutpansberg Mountains travelled through a dense route network, habitually using the same tracks, a pattern of navigation that has been reported for a number of other primate species (Boonratana 2000; Di Fiore and Suarez 2007; Erhart and Overdorff 2008; Hopkins 2011; Mackinnon 1974; Presotto et al. 2018; Schreier and Grove 2014; Trapanese et al. 2018). In primates, habitual routes often coincide with streams, ridges of hills and tracks located in their home range (Di Fiore and Suarez 2007; Mackinnon 1974). We also observed our study group frequently making use of man-made tracks and game trails and the identified ‘highway’ network often overlapped with these (Fig. 2c). Game trails and man-made tracks may be used as landmarks themselves and are often highly linear. The use of roads to orientate travel has also been reported for other baboon populations (Noser and Byrne 2014).

The use of habitual travel routes is not necessarily evidence that primates navigate (solely) using a topological map or that they lack a Euclidean spatial representation (Noser and Byrne 2007a; Presotto and Izar 2010). Indeed, contrary to the prediction for the topological map, the linearity index for our group was not significantly different between the core home range and peripheral areas, regardless of distance travelled between consecutive travel goals. Although perfect linearity of travel routes is unlikely and would not necessarily be the optimal route in natural habitats, the linearity of the travel routes in the core area and the periphery was not particularly high (median linearity ratio: 0.82 and 0.81 respectively) compared to other studies on primate travel routes. 78.5% and 72.4% of the path segments in the core area and periphery, respectively, were considered ‘highly linear’, *i.e*. with linearity ratios above 0.7 (Valero and Byrne 2007, p. 310). In comparison, Noser and Byrne (2007b) found a median linearity ratio of 0.88 and 44% of segments reached a linearity ratio between 0.9 and 1.0 in chacma baboons at the nearby Blouberg Nature Reserve, while Valero and Byrne (2007) reported 78% of route segments had linearity ratios above 0.8 in spider monkeys. Normand and Boesch (2009), who argued that chimpanzees (*Pan troglodytes*) possessed Euclidean-based spatial awareness, found an average linearity ratio of 0.96. Therefore, although baboons in the Soutpansberg Mountains did not travel more directly in the core area than in the periphery, potentially indicating Euclidean spatial awareness, linearity throughout the home range was not as high as one might expect under the hypothesized use of a Euclidean map. Furthermore, although our baboon group used significantly more direct travel in winter when food resources were scarce such that cognitive mechanisms may become more evident (Valero and Byrne 2007), there was no difference in the linearity of path segments between core and peripheral areas. It is thus likely that chacma baboons in the Soutpansberg Mountains have accumulated a similar knowledge of the periphery as of the core area, which allowed them to navigate with similar efficiently in both areas.

If our baboon group was using a Euclidean map rather than a topological map, we predicted they would approach goals from all different directions, whereas when using a topological map they were predicted to approach their travel goals from consistent direction(s). We found approach directions to 80% of travel goals were significantly clumped (deviated from a random circular distribution) and did not differ by season, indicating the consistent use of routes that is more in line with the hypothesized use of a topological map. This contrasts with evidence for some primate species that approach resources from many different directions. Saddle-back (*Saguinus fuscicollis*) and moustached tamarins (*Saguinus mystax*) approached 15 preferred trees from all directions (Garber 1986), while approach angles for chimpanzees revisiting resources were not consistent with simulated approach angles based on network routes (Normand and Boesch 2009). Both studies used linear statistical analysis of angular data, however, and circular statistics designed for directional data could produce different outcomes (de Raad 2012). Future studies should be mindful of this potential impact on interpretation and ensure that angular data are analysed using appropriate statistics (Mardia and Jupp 2009).

If our study group were using Euclidean spatial awareness rather than topological spatial awareness, we predicted the baboons would initiate navigation to a resource in a direction consistent with the actual direction of that resource (Normand and Boesch 2009). In contrast, if the baboons were using landmarks to orient themselves they would show greater deviation from this goal-directed travel (following Di Fiore and Suarez 2007), a prediction that was supported by our analyses. Since this finding also did not vary with season this again indicates that baboons in the Soutpansberg Mountains do not use Euclidean spatial awareness. The use of a topological map in an environment with sufficient landmarks should result in highly efficient and direct travel without the need for Euclidean spatial awareness (Byrne 2000) and it is thus likely that in a mountainous area with many prominent landmarks, such as cliffs and mountain peaks, the baboons were able to navigate efficiently without using Euclidean navigation. Such a conclusion, however, cannot dismiss the baboons’ potential ability to calculate novel routes in the absence of such landmarks or that baboons lack Euclidean spatial awareness all together. Indeed, the fact the group was highly familiar with its local environment and had traversed its familiar routes multiple times may ultimately preclude definitive conclusions and experimental approaches might be needed to create unfamiliar situations. Nevertheless, our results suggest that baboons within the Soutpansberg Mountains use route-based navigation, a topological map, to travel towards goals and we have found no evidence of our baboons making use of Euclidean spatial awareness.

Normand and Boesch (2009) suggested the potential for Euclidean spatial representation in chimpanzees, though this finding has been challenged (Trapanese et al. 2018). In theory, Euclidean spatial awareness is characterised by greater flexibility and efficiency and in the anthropological sciences it has been argued that Euclidean spatial abilities were selected for due to the challenge of maintaining complex, spatially extensive social networks (Ambrose 2001; Burke 2012; Hartley et al. 2007; Leonard and Robertson 2000; Maguire et al. 2003; Potts 2004). Specifically, Potts (2004) has argued the recurrent parting and joining of individuals in fission–fusion societies increased the potential for evolving representational intelligence, including a theory of mind and self-conception. While fission–fusion social organisation offers flexibility to cope with ecological uncertainty and food dispersion, the problems posed by temporally and spatially distant entities (including group members) enhance the potential for cognitive evolution. While chimpanzees live in fission–fusion societies where individuals forage in small, temporary parties or sub-groups that change in size and composition, spider monkeys live in similar communities (Di Fiore and Campbell 2007) but did not display Euclidean spatial awareness in large-scale space (Di Fiore and Suarez 2007). Whether there is some threshold of social complexity in primates that may act as a selective pressure for Euclidean cognitive abilities is a question that requires further research.
